# Composite Binder Containing Industrial By-Products (FCCCw and PSw) and Nano SiO_2_

**DOI:** 10.3390/ma14071604

**Published:** 2021-03-25

**Authors:** Vilma Banevičienė, Jurgita Malaiškienė, Jiri Zach, Karel Dvorak

**Affiliations:** 1 Laboratory of Composite Materials, Faculty of Civil Engineering, Institute of Building Materials, Vilnius Gediminas Technical University, 10223 Vilnius, Lithuania; vilma.baneviciene@vilniustech.lt; 2 Faculty of Civil Engineering, Institute of Building Materials and Components, Brno University of Technology, 60200 Brno, Czech Republic; zach.j@fce.vutbr.cz (J.Z.); dvorak.k@fce.vutbr.cz (K.D.)

**Keywords:** composite binder, catalyst waste (FCCCw), paper sludge waste (PSw), nano SiO_2_ (NS), properties

## Abstract

This article analyzes the integrated effect of industrial by-products (spent fluidized bed catalytic cracking catalyst waste (FCCCw) and paper sludge waste (PSw) generated in paper manufacturing) combined with nano-SiO_2_ (NS) on the properties of cement binder, when a certain part of the binder is replaced with the said by-products in the cement mix. Standard testing methods were used to analyze the physical and mechanical properties of cement-based materials. For structure analysis, we used X-ray diffraction (XRD), derivative thermogravimetry (DTG), mercury intrusion porosimetry (MIP) and scanning electron microscopy (SEM). It was found that the replacement of cement by a combined additive of FCCCw, PSw and NS is important not only for ecological reasons (abatement of CO_2_ emissions and recovery of waste through secondary raw materials), but also in order to enhance the properties of cement-based binders. Presumably, higher amounts of calcium silicate hydrate (CSH) and calcium alumina silicate hydrate (CASH) in the compound binder are the result of the low content of portlandite and alite in the test specimens. The specimens modified with all three additives had the highest density (~2100 kg/m^3^), ultrasonic pulse velocity (UPV) (~4160 m/s) and compressive strength (~105 MPa), which was ~40% higher than in the control specimens. The average pore diameter of the complex binder decreased by 21%, whereas the median pore diameter decreased by 47%.

## 1. Introduction

Currently, the issue of CO_2_ abatement in the cement industry is very important. Greenhouse gas emissions could be reduced by reducing clinker production and by replacing clinker in cement with some industrial waste, which would also add value to the development of the circular economy. It is also an important goal for the clinker substitutes used to provide better properties in cement mixes used for different applications. For instance, water-permeable concrete requires fast-curing and lower free calcium binders because water-permeable concrete structures are constantly exposed to running water, which gradually washes out Ca(OH)_2_ and reduces the durability of the concrete. Additionally, a high content of portlandite in cementitious materials should be minimized, because it decreases the durability and compressive strength of concrete [1,2]. Portlandite is the most soluble mineral and can dissolve by increasing the porosity of concrete or mortar [3]. To avoid such negative effects, pozzolanic additives (for example, from industrial waste) are usually used, or epoxy resins may be used to protect cementitious coatings [4].

Petrochemical industries all over the world generate hundreds of thousand tons of spent catalysts annually [5]. Scientists have provided data on the high pozzolanic activity of the catalyst used in the fluidized-bed catalytic cracking units of oil refineries and the possibilities of using the spent fluidized bed catalytic cracking catalyst waste (FCCCw) in cement-based materials [6]. Thermogravimetric analysis (TGA) showed that calcium alumina hydrate (CAH) and calcium alumina silicate hydrate (CASH) contents were higher in cement samples with FCCCw [6,7]. The pozzolanic activity of FCCCw was confirmed by the increased compressive strength values of the specimens in which a part of cement was replaced by FCCCw [8,9]. FCCCw with high pozzolanic activity can increase the resistance to the alkali-aggregate reaction [10], reduce porosity with the filling effect and increase the strength [11,12] of cement-based materials. Authors have also noted [13] that the optimal amount of cement to be replaced by the aforementioned materials ranges between 15–20%. A research article [14] reports that the compressive strength of a composite material produced from ecological cement containing 4% of FCCCw was similar to the strength of ordinary Portland cement (OPC). Another study [15] reported higher heat of hydration values observed in concrete mixes where cement was substituted by 10% of FCCCw (the paper provides test results for specimens modified with 0%, 10% and 35% of FCCCw). Authors [16] have also found that FCCCw generated in oil refineries in Portugal has pozzolanic properties and can be used in the manufacture of ecological structural concrete. According to the findings reported in other research articles [17,18,19], the FCCCw used in the tests definitely had high pozzolanic activity, whereas the binder containing FCCCw demonstrated a higher compressive strength and resistance to chemical reactions.

The high volume of pulp and paper mill sludge (PSw) is a global problem faced by all countries. The European paper industry generates 11 million tons of paper sludge per year [20], whereas the annual volume of paper sludge generated in Korea reaches 26 million tons [21,22,23]. Due to high (~50%) content of CaO, the presence of calcite and the uniform distribution of grain sizes, paper sludge is a promising secondary raw material in the construction industry, offering the potential improvement of mechanical properties and technological characteristics of construction materials [24]. Up to 10% of PSw, which is rich in lime, can be added to cement-based mortars used for external applications [25]. Moises Frias et al. [26] recommend applying a calcination process at 650 °C for 2 h. According to their test results, the organic part of the material was totally decomposed and kaolinite was transformed into metakaolinite. A research paper [27] reports that chemical, physical and mechanical characteristics of blended cements containing calcined PSw satisfy the requirements prescribed by the European standard 197-1. Therefore, it is feasible to incorporate calcined PSw as an active additive in cement manufacturing. After 7 days of curing, a significant gain in compressive strength (approximately 10%) was observed in specimens in which 10% of calcined paper sludge was blended with OPC (CEM I). Another research paper [28] reports that calcination is not required and it is sufficient to dry the PSw at 75 °C temperature. Up to 5% of dried PSw can be added to the mortar mixes by weight of cement or a natural fine filler to reduce the amount of cement used and to improve the properties of the mortar. The authors of that article state that dried PSw delayed cement hydration. However, the hydration rate increased and the physical-mechanical properties of cement-based materials improved (e.g., the compressive strength of the modified specimen was ~13% higher than the strength of the control specimen) when PSw was heated at 700 °C for 2 h [23]. A negative effect of PSw in lightweight calcium silicate board materials was reported [29] due to reduced water absorption in the tested boards. PSw can be used as an active additive and also as a filler when it is pre-treated with sodium hydroxide. Mortars modified with pre-treated PSw demonstrated better mechanical and durability properties. The compressive strength at 50 days of the tested mortars increased up to 20% [30]. PSw used along with recycled aggregates in concrete was found to increase the compressive strength and resistance of concrete to acid and sulphate attacks [22]. The important test results presented in [31] show that PSw can be added to mortar mixes in a liquid state; however, far better physical and mechanical properties were obtained with calcinated PSw.

Nanomaterials as cement substitutes are being extensively studied for their application in the production of building materials. Nano SiO_2_ particles (NS) can act as pozzolanic additives in cement-based materials to make the cement matrix denser and to reduce the size of micro pores [32,33,34]. Another paper [35] presents the analysis of the effect of colloidal nano-silica (CNS) dosed at 0.5%, 1.0%, 1.5% and 2.0% on the properties of cement-based materials. The structure of the specimens containing CNS was denser and more homogeneous because of pozzolanic reactions. CNS was also found to accelerate cement hydration due to the nucleating effects. More calcium silicate hydrate (CSH) was produced in the reaction of CNS and Ca(OH)_2_, leading to lower porosity of the specimens, development of smaller pores and higher compressive strength. Researchers [36] have proposed combining micro-silica and nano-silica for a better synergistic effect and higher effectiveness of the modification. According to the test results, the specimens modified both with micro-silica and nano-silica had the best mechanical characteristics. It has been reported [37] that higher NS doses increased the heat of hydration at 1 day and 3 days, and increased the compressive strength at 1 day, but reduced the compressive strength at 3, 7 and 28 days. In another paper [38], it was also reported that NS increases the rate of cement hydration and the heat of hydration, which, in turn can increase shrinkage. NS was found [39] to increase the hydration rate in the early stage due to faster dissolution of cement and the nucleation of the hydrate on the reacted NS particles. A higher content of NS accelerated the hydration of alite at 3 days, but had a retarding effect at 7 days, 28 days and 60 days [40]. The amount of calcium silicate hydrate increases with the higher content of NS [41]. Nano silica influences cement hydration immediately after contact with water [42].

The effects of FCCCw, PSw and NS on the properties of cement-based materials have been analyzed by researchers; however, no studies have been found analyzing the properties of cement-based materials modified by all three additives simultaneously. The novelty of this study and the aim of this work is to analyze the combined impact of FCCCw, PSw and NS on the structure and physical-mechanical properties of cement-based binder. It was found, that: (1) the replacement of 12.5% of cement by industrial waste (FCCCw and PSw) is important for ecological reasons, as it decreases amount of clinker required in the cement paste; (2) the new complex binder (cement + NS + PSw + FCCCw) had the lowest amounts of portlandite and alite, presumably due to the higher amounts of CSH and CASH; (3) the compressive strength of the new complex binder was ~40% higher compared to the control specimens; (4) the densest structure was established in the new complex binder specimens.

## 2. Materials and Methods

### 2.1. Characteristics of Materials

Cement CEM I 42.5 R (Rocket cement M-600, HeidelbergCement, Skovde, Sweden) satisfying the requirements of EN 197-1 was used for the tests. The chemical composition of the cement (weight, %) was as follows: CaO 63.2; SiO_2_ 20.4, Al_2_O_3_ 4.0, Fe_2_O_3_ 3.6, MgO 2.4, K_2_O 0.9, Na_2_O 0.2, SO_3_ 3.1, Cl 0.05, L.O.I. 2.15. The mineral composition of the cement used was as follows: C_3_S—56.6%, C_2_S—16.7%, C_3_A—9.0%, C_4_AF—10.6% and 7.1% other substances. Other parameters of the cement used: specific density 3.1 g/cm^3^, compressive strength at 28 days 55 MPa, initial setting time 180 min.

The FCCCw used for the tests was the production residue from AB Orlen Lietuva oil refinery (Mazeikiai, Lithuania). The chemical composition of FCCCw (weight, %) was as follows: SiO_2_—50.1; Al_2_O_3_—39.4; SO_x_—2.30; Fe_2_O_3_—1.30; CaO—0.50; MgO—0.49; Na_2_O—0.20; K_2_O—0.07; Mn_2_O_3_—0.06. FCCCw particles were spherical and the average diameter was ~40 μm. Pozzolanic activity of FCCCw—1017 mg/g (NF P18-513 Chapelle test). According to the XRD results FCCCw was faujasite (F), i.e., the Y type of zeolite. There are two types of zeolites: X and Y. Zeolite Y has a higher Si/Al atomic ratio than zeolite X and has higher thermal stability. Such Y zeolites are used as catalysts in oil manufacturing [43].

NS properties: purity 99.8%, particle surface area 202 m^2^/g, pH (40 g/L) 4.0, relative density 2.2 g/cm^3^, size 10–30 nm (Sigma-Aldrich Chemie GmbH, Merck, Taufkirchen, Germany). The pozzolanic activity of NS—1695 mg/g (according to NF P18-513 Chapelle test), much higher than the activity of FCCCw.

Dried PSw (AB Grigeo, Vilnius, Lithuania) was burnt at 700 °C for 2 h. The size of PSw particles <0.1 mm. XRD analysis revealed that in PSw, calcinated at 700 °C, calcite and calcium oxide were identified. The chemical composition of PSw (weight, %) was as follows: SiO_2_—5.1; Al_2_O_3_—3.8; SO_x_—0.4; Fe_2_O_3_—0.6; CaO—88.0; MgO—1.2; Na_2_O—0.20; K_2_O—0.08.

Superplasticizer Melment F10 (SP) is a free-flowing spray-dried powder of a sulphonated polycondensation product based on melamine (BASF Construction Polymers GmbH, Trotsberg, Germany), pH 9.4. Tap water was used for compositions, pH 7.8.

Specimens (160 × 40 × 40 mm) were prepared by replacing a part of cement with FCCCw, PSw and NS. Compositions of the cement-based binder are presented in Table 1. The 0.02% amount of NS was chosen according our previous work, which analyzed the impact of NS types and amount from 0.01% up to 0.3% [44]. The best results of physical mechanical properties of hardened cement paste with PSw were established by replacing 2.5 of cement [45], and by replacing 10% of cement with FCCCw [5], using them separately.

Initially the NS was dispersed in water (100 mL) for 5 min at 400 W 22 kHz by means of an ultrasonic disperser UZDN-2T (RKPO, Moscow, Russia). The superplasticizer was added to the prepared suspension. The temperature of suspension was 20 °C ± 2 °C. The cement-based binder was mixed in the Hobart mixer for 2 min according to EN 196-1 and poured into 160 mm × 40 mm × 40 mm molds.

### 2.2. Methods

The specimens were unmolded after 1 day and cured until the testing date at the 7th and 28th day of curing according to standard EN 196-1. Three specimens of each curing age were tested for density and UPV and six specimens were tested for compressive strength. The density of the specimens was established according to the procedure described in standard EN 196. Ultrasonic pulse velocity (UPV) was determined according to the literature [45]. The compressive strength of the specimens hardened in water for 7 and 28 days was measured by means of a hydraulic press, ALPHA3-3000 S, according to EN 196.

Powder X-ray diffraction analysis was conducted on a Panalytical Empyrean diffractometer (Malvern Panalytical, Almelo, The Netherlands) equipped with a Cu-anode and a 1-D position-sensitive detector at convention Bragg–Brentano reflection geometry. The settings were: step size—0.013 2θ, time per step—158 s, angular range 5–80 2θ, voltage—45 kV; current—40 mA. Quantitative phase analysis was done via the Rietveld method using Panalytical High Score 3 plus software and the ICSD database (release 2012). The amorphous phase content of the sample was estimated using the “constant background intensity” method, in which the crystallinity of a sample is defined as the intensity ratio of the diffraction peaks and of the sum of all measured intensity—it can be calculated using an Equation (1):

(1) $$\mathrm{Cryst}=\frac{100\cdot \sum I_{\mathrm{net}}}{\sum I_{\mathrm{tot}}-\sum I_{\mathrm{const}.\;\mathrm{bgr}.}}$$

where Cryst is crystallinity, %, I_net_ is the area of the crystalline peaks, I_tot_ is total area and I_const.bgr._ is the area of the constant background. Note that even a completely crystalline sample has some background intensity, which arises from imperfections of the sample, the X-ray optics of the instrument, sample fluorescence and scatter. This constant background intensity is subtracted from the total intensity [46]. The background intensity was determined by separating all the crystalline peaks. The amorphous phase content calculations were performed using the Panalytical Highscore + 3 program.

Derivative thermogravimetric analysis (DTG) was performed using a Mettler Toledo TGA/DSC 1 device (Switzerland). Samples with a mass of 50–60 mg were placed in a platinum crucible and heated at 10 °C/min in a nitrogen environment up to 1000 °C. The amount of portlandite, %, was calculated according to the mass loss in the 430 °C–560 °C temperature range (m_H2Ol_). Then, the amount of decomposed portlandite (m_Pd_) was calculated from the Equation (2):

(2) $$m_{\mathrm{Pd}}=m_{H2\mathrm{Ol}}\cdot \frac{R_{P}}{R_{w}}$$

where m_H2Ol_ is mass loss in the 430 °C–560 °C temperature range, Rp is the relative molecular weight of portlandite and Rw is the relative molecular weight of water. Afterwards, the amount of portlandite m_Pdr_ on a dry basis and the amount of portlandite mc on a cement basis were calculated from Equations (3) and (4):

(3) $$m_{\mathrm{Pdr}}=\frac{m_{\mathrm{Pd}}}{m_{600}}\cdot 100,\%$$

(4) $$m_{c}=\frac{m_{\mathrm{Pdr}}}{K}$$

where m_600_ is the mass loss at 600 °C temperature, m_Pd_ is the calculated amount of decomposed portlandite and K is the coefficient depending on the amount of cement in the binder. The mercury intrusion porosimetry (MIP) method was applied to analyze the parameters of hardened cement paste pore structure by means of a Quantachrome Poremaster 33/60 (Quantachrome Instruments, Boynton Beach, FL, USA) with a maximum pressure of 33,000 psi for pore diameters ranging from 1100 μm to 0.0035 μm and with two low-pressure stations plus one high-pressure station.

The microstructure of the hardened cement pastes was observed by means of scanning electron microscopy (SEM) in secondary electron (SE) mode using a JEOL JSM-7600F device (JEOL Ltd., Tokyo, Japan). The images were obtained from gold-coated surface fractures of the hardened samples using the vacuum evaporation technique. The following microscope settings were used: 10 kV and 20 kV voltage; 7–10 mm distance to sample surface.

## 3. Results and Discussion

### 3.1. Results of XRD Analysis

**Figure 1:**
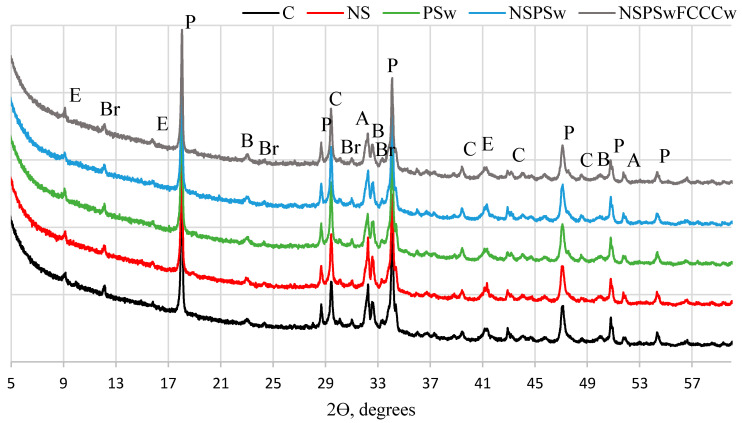
XRD pattern of hardened cement paste at 7 days.

**Figure 2:**
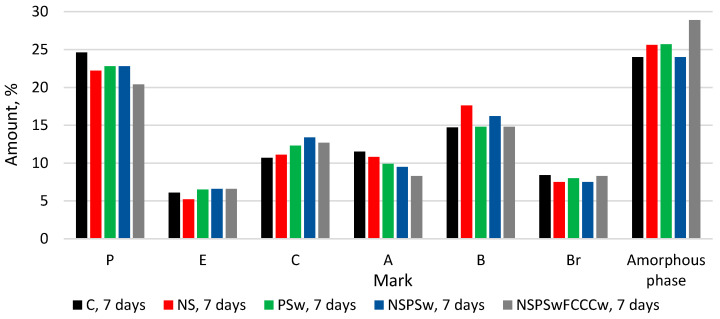
The comparison of the amount of minerals and amorphous phase at 7 days.

**Figure 3:**
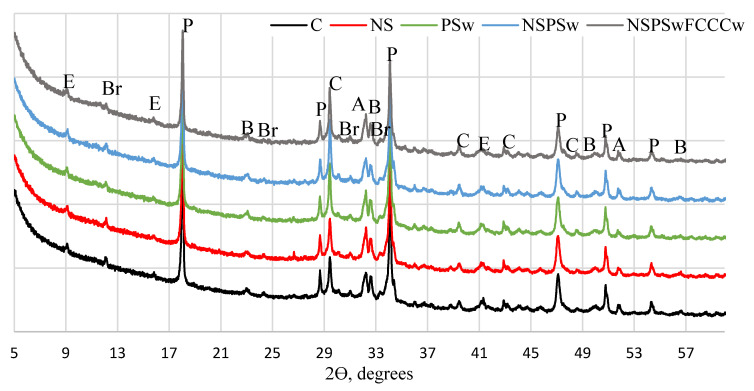
XRD pattern of hardened cement paste at 28 days.

**Figure 4:**
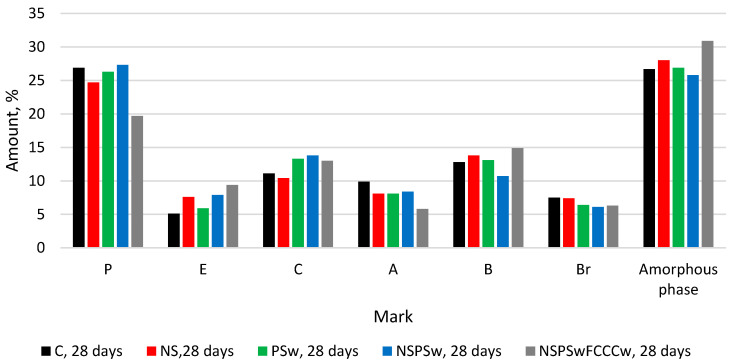
The comparison of the amount of minerals and amorphous phase at 28 days.

The same minerals in all compositions were identified in XRD diffractograms (Figure 1, Figure 2, Figure 3 and Figure 4) of the specimens containing different additives of NS, PSw and FCCCw at 7 and 28 days of curing: portlandite P (ref. code 98-006-4950), ettringite E (ref. code 98-002-7039), calcite C (ref. code 98-004-0107), alite A (ref. code 98-002-2501), belite B (ref. code 98-007-9551) and brownmillerite Br (ref. code 98-009-7926). Relative changes of portlandite, ettringite, calcite, alite, belite, brownmillerite and amorphous phase were analyzed according to the Rietveld method (Figure 2 and Figure 4). The amount of portlandite was found to decrease with the reduction of the cement content by replacing it with pozzolanic materials (NS, PSw and FCCCw), irrespective of the curing time. The amount of portlandite decreased because of the cement dilution effect and intensive pozzolanic reactions of the additives. The lowest amount of portlandite was found in the specimens containing all three additives. Compared to the control specimens, the amount of portlandite decreased by 17% at 7 days and ~27% at 28 days. Pozzolanic additives reduced the amount of initial cement mineral—alite and more amorphous phases calcium silicate hydrate (CSH) and calcium alumina silicate hydrate (CASH) were formed. The lowest amount of alite was found in the specimens with the NS/PSw/FCCCw composition, in which all three pozzolanic additives were combined. Compared to the control specimens, at 7 days the amount of alite in these specimens decreased ~28% and at 28 days the amount of alite decreased ~40%. The amount of amorphous phase of NS/PSw/FCCCw compared to control specimens increase 15–20%. A significant increase in calcite was observed in the specimens containing PSw. PSw is rich in calcium, which presumably was transferred to the cement specimens. The change in the amounts of belite and brownmillerite in these compositions was not so significant, but the amount of ettringite using such pozzolanic additives can increase with time.

### 3.2. Results of DTG Analysis

**Figure 5:**
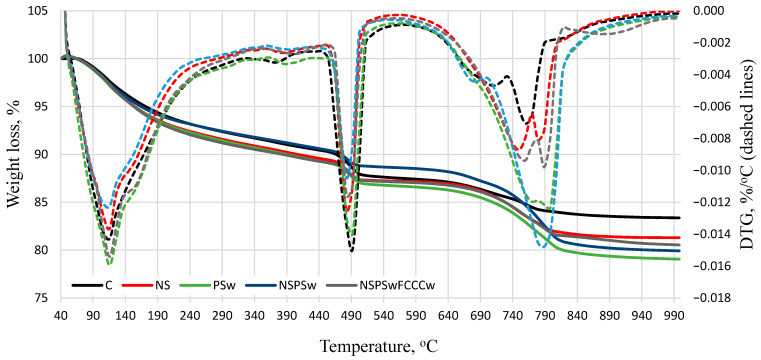
Results of derivative mass loss rate and cumulative mass loss at 7 days.

**Figure 6:**
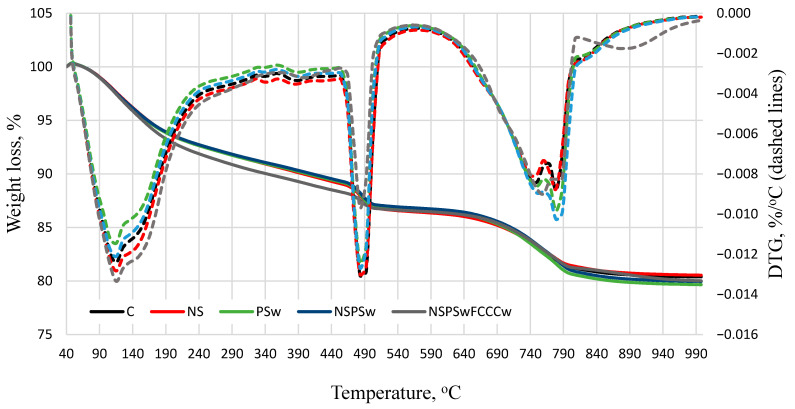
Results of derivative mass loss rate and cumulative mass loss at 28 days.

The results of the thermographic analysis presented in Figure 5 and Figure 6 and Table 2 show that with the temperature rise from 25 °C to 1000 °C, three major peaks are formed in the following temperature ranges: 70 °C–190 °C, 440 °C–540 °C and 690 °C–850 °C. According to literature [47], most of the evaporable water is removed in the range of 30 °C–105 °C; the decomposition of ettringite, the loss of a portion of water of CASH and CSH, and the removal of all evaporable water occurs in the range of 110 °C–170 °C; the loss of the remaining part of the CSH, especially the CASH, occurs at 180 °C–350 °C; the dehydroxylation of portlandite occurs at ~430 °C–560 °C; and the decarbonation of calcium carbonate occurs at ~690 °C–850 °C.

After 7 days of curing, the biggest mass loss in the temperature range up to 350 °C was observed in the NS/PSw/FCCCw specimens. Compared to the control specimen, the mass loss was ~14% higher. After 7 days of curing, the lowest amount of portlandite was determined in NS/PSw/FCCCw specimens. The highest amount of calcite was determined in specimens containing PSw due to the calcite present in the material added to the mix. The highest amounts of portlandite and the lowest amounts of calcite were observed in the control specimens both after 7 and 28 days of curing. The amount of portlandite decreases when a portion of cement is replaced with pozzolanic additives. Presumably, more CSH and CASH are formed in the reaction of portlandite with active SiO_2_ present in the additives. Similar trends were observed after 28 days of curing—the biggest mass loss in the temperature range up to 350 °C was observed in the specimens containing the compound binder additive NS/PSw/FCCCw. Presumably, the mass loss indicates higher amounts of CSH and CASH in this composition because the lowest amount of portlandite was also observed in the specimens containing pozzolanic additives. The results of the thermographic analysis are similar to XRD results and suggest that the compound binder will produce the best physical and mechanical properties because CSH has a significant influence on the mechanical strength of cementitious materials due to van der Waals interactions, which ensure the cohesion and adhesion between the solid phases [48].

### 3.3. Results of Physical Mechanical Properties

**Figure 7:**
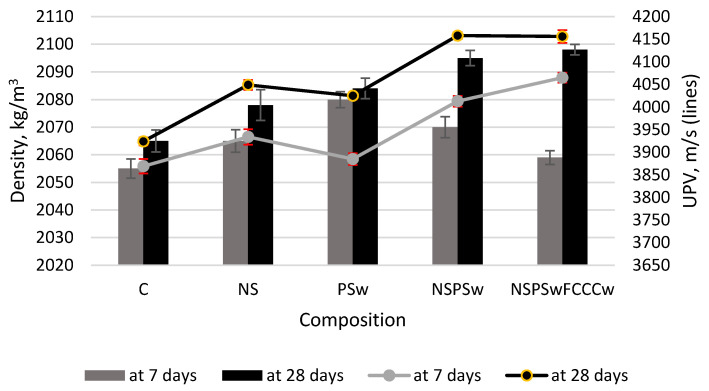
Density and ultrasonic pulse velocity (UPV) values of hardened cement paste at 7 and 28 days.

Figure 7 illustrates the density and ultrasonic pulse velocity in the specimens. The obtained results revealed that after both 7 and after 28 days of curing, the density and UPV values of the modified specimens were higher than the same values of the control specimens (UPV shows how the structure of the material is formed and compacted). This increase can be explained by the pozzolanic activity and the filling effect of the additives. The highest UPV values were recorded in the specimens modified with a combined binder NS/PSw/FCCCw. After 28 days of curing, the density of this composition compared to the control specimen increased ~2% (from 2065 kg/m^3^ to ~2100 kg/m^3^), and the UPV increased ~6% (from 3920 m/s to 4160 m/s).

**Figure 8:**
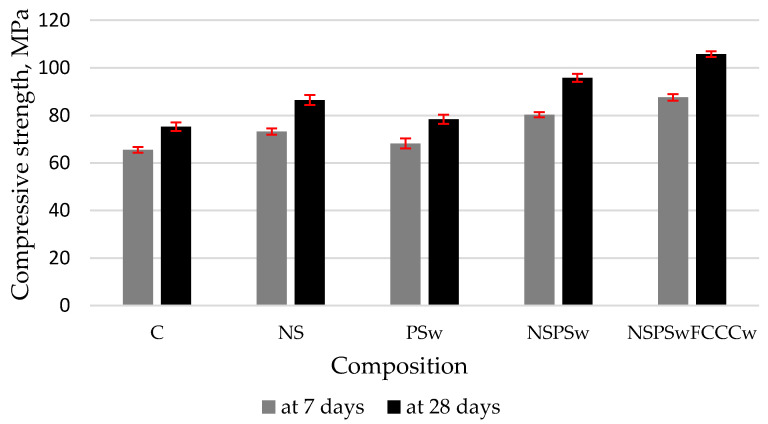
Compressive strength of hardened cement paste at 7 and 28 days.

**Figure 9:**
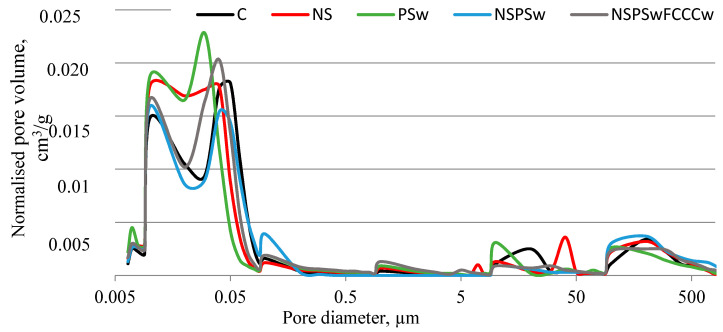
Results of normalized pore volume in hardened cement paste.

**Figure 10:**
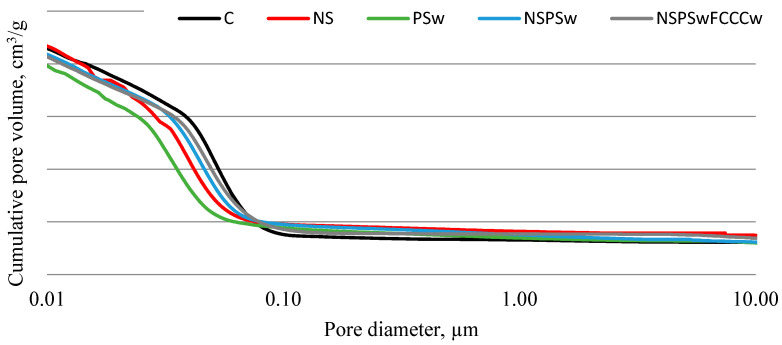
Results of cumulative pore volume in hardened cement paste.

The results of compressive strength tests (Figure 8) showed the most significant changes when the compound binder was used. The compressive strength of the specimen modified with NS increased ~13.5% compared to the control specimen. An increase of ~25% was observed when the mix was additionally modified with PSw. The replacement of cement with 10% of FCCCw gave a ~40% increase in compressive strength at 28 days. The increase in compressive strength was caused by the following physical processes: the decrease of porosity and pore size (Figure 9 and Figure 10), higher density and UPV values (Figure 7), as well as chemical processes, namely the pozzolanic reaction, which activated the hydration (XRD, DTG analysis results) and presumably resulted in higher amounts of CSH.

### 3.4. Results of MIP and SEM Analysis

The porosity analysis (Figure 9 and Figure 10, Table 3) produced pore size distribution curves and results in terms of accessible porosity, total porosity, total surface area and pore diameters (average, median). All compositions had a bimodal pore size distribution with the first peak at ~0.01 µm and the second peak with higher intensity at ~0.04 µm. The lowest total porosity and the smallest average and median pore diameters, as well as some of the lowest accessible porosity, were determined in the specimens modified with a combined binder containing NS, PSw and FCCCw. Compared to the control specimens the average pore diameter decreased by 21%, and median pore diameter was 47% smaller. A larger decrease in the pore diameter was observed in the case of NS, with the strongest pozzolanic characteristics. An even greater effect was observed when this additive was used in combination with other pozzolanic additives. The decrease in micro-porosity caused by the pozzolanic additives can be explained by the greater amounts of CSH and CASH produced and the filling effect of finer additives.

**Figure 11:**
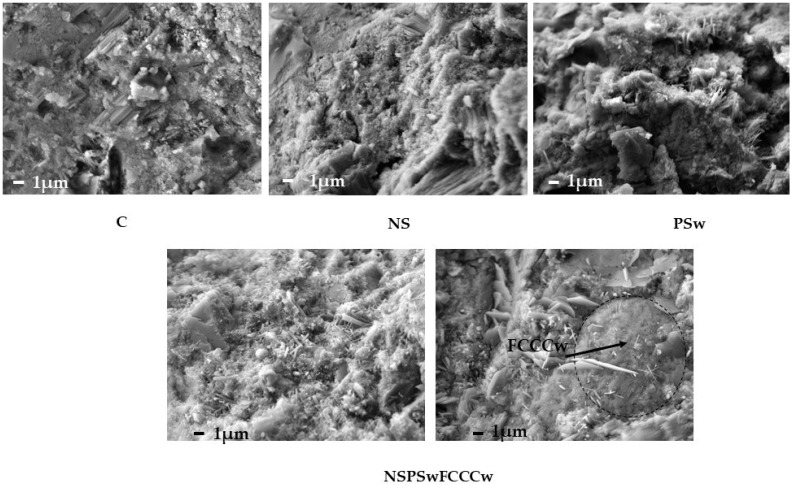
Microstructure images of the most typical batches (magnification 5000 times).

SEM images (Figure 11) showed that all specimens had a dense and uniform structure. The most porous structure is visible in the control specimens and the specimens modified with PSw. The densest structure was fixed with the specimens with the complex binder (NS + PSw + FCCCw). A FCCCw particle with the build-up of the standard cementitious structure on and around it is shown in the image of the NS/PSw/FCCCw microstructure. There was a strong connection between FCCCw particles and the cement matrix.

## 4. Conclusions

It was found that the replacement of cement by a combined additive of FCCCw, PSw and NS was effective not only for ecological reasons (abatement of CO_2_ emissions and recovery of waste through secondary raw materials) but also for enhancing the properties of cement-based binders. Pozzolanic additives must be used in concrete structures exposed to water because of Ca(OH)_2_ leaching. XRD and DTG analyses revealed that the lowest amount of portlandite was formed in the specimens modified with a combined binder. The amount of portlandite continued decreasing when PSw and later FCCCw were added to the cement mix. The NS/PSw/FCCCw binder had the lowest amounts of portlandite and alite, presumably due to the higher amounts of CSH and CASH.

The specimens modified by all three additives (NS, PSw, FCCCw) at the same time had the highest density (~2100 kg/m^3^), UPV (4160 m/s) and compressive strength (~105 MPa) values. After 28 days of curing, the compressive strength of NS/PSw/FCCCw specimens increased ~40% compared to the control specimens. The increase in compressive strength was only 13.5% when the mix was modified with NS only.

Compared to the control specimens, the average pore diameter decreased by 21%, and the median pore diameter was 47% smaller. A more significant decrease in the pore diameter was observed in specimens modified with NS, which had the strongest pozzolanic characteristics. A stronger diminishing effect in the pore size was observed in the specimens additionally modified with PSw and FCCCw. SEM analysis results revealed an intensive build-up of crystallized hydrates on and around FCCCw particles, creating strong links with the cement matrix.

## Figures and Tables

**Table 1:** Compositions of cement-based binder.

Composition	Cement, %	NS, %	PSw, %	FCCCw, %	Superplasticizer, % (up to 100%)	Water/Binder
Control (C)	100	–	–	–	0.2	0.32
nano SiO_2_ (NS)	99.98	0.02	–	–	0.2	0.32
paper sludge waste (PSw)	97.5	–	2.5	–	0.2	0.32
NS/PSw	97.48	0.02	2.5	–	0.2	0.32
NS/PSw/fluidized bed catalytic cracking catalyst waste (FCCCw)	87.48	0.02	2.5	10.0	0.2	0.32

**Table 2:** Mass loss in decomposition temperature ranges at 7 and 28 days.

Mark	110 °C–170 °C	180 °C–350 °C	430 °C–560 °C	Amount of Dry Basis Portlandite, %	Amount of Cement Basis Portlandite, %	690 °C–850 °C
C7	2.8	3.0	3.0	14.0	14.0	2.6
NS7	3.2	3.0	2.4	11.5	11.5	4.6
PSw7	3.2	3.2	2.7	12.6	12.9	5.8
NS/PSw7	2.8	2.7	2.3	10.9	11.2	6.7
NS/PSw/FCCCw7	3.4	3.2	2.2	10.5	11.9	4.7
C28	3.2	3.4	2.9	14.0	14.0	4.4
NS28	3.2	3.4	2.9	13.8	13.8	4.2
PSw28	3.2	3.3	2.9	13.6	13.9	5.2
NS/PSw28	3.1	3.2	2.8	13.2	13.5	5.1
NS/PSw/FCCCw28	3.6	3.7	2.0	9.7	11.0	4.4

**Table 3:** Mercury intrusion porosimetry (MIP) results in terms of pore diameters (average, median), total surface, accessible and total porosity.

Characteristic	C	NS	PSw	NS/PSw	NS/PSw/FCCCw
Average pore diameter, µm	0.032	0.027	0.031	0.029	0.025
Median pore diameter, µm	0.050	0.039	0.050	0.044	0.033
Total pore surface area, m^2^/g	15.01	17.65	15.13	16.61	18.35
Accessible porosity, %	19.92	19.79	18.60	19.36	18.98
Total porosity, %	23.02	23.73	22.47	22.70	22.21
